# Surveillance and Surgery for IPMN: An Urgent Call to Challenge the Status Quo!

**DOI:** 10.1002/ueg2.70232

**Published:** 2026-05-27

**Authors:** Sanjay Pandanaboyana, Kjetil Soreide

**Affiliations:** ^1^ Hepatobiliary and Pancreatic Unit Freeman Hospital Newcastle Upon Tyne UK; ^2^ Population Health Sciences Institute Newcastle University Newcastle UK; ^3^ Department of Surgery Institute of Clinical Sciences Sahlgrenska Academy University of Gothenburg Gothenburg Sweden; ^4^ Department of Surgery Sahlgrenska University Hospital Gothenburg Region Västra Götaland Sweden

**Keywords:** cyst, pancreas, surveillance

Pancreatic cystic lesions (PCL) are an increasing clinical challenge to clinicians and patients alike. Their prevalence is estimated to be around 16% in patients undergoing cross‐sectional abdominal imaging for unrelated indications, discovered incidentally [1]. While a large number of such incidental PCL may be small (< 1 cm) and of indeterminate aetiology, approximately 80% of all PCLs are classified as intraductal papillary mucinous neoplasms (IPMNs), mostly of the branch‐duct type. As IPMN is a recognized precursor to pancreatic cancer, detection of such lesions warrant surveillance according to current guidelines. Image guided surveillance for IPMN has been shown to result in psychological impact on patients, resulting in “scanxiety” before new imaging, in addition to an increasing financial burden to health care systems from the cost of surveillance programs. In this Journal, two recent multicenter studies reported on surveillance for small and stable pancreatic cysts [2] and survival outcomes after pancreatic resection [3] following preventive resection respectively. Both studies point to considerable challenges with the status quo for managing PCLs, with overuse of surveillance and poor accuracy in predicting high‐risk lesions in patients being considered for surgery.

Levink et al. [2] investigated the risk of developing high grade dysplasia (HGD) and pancreatic cancer (PC) during follow‐up for different cyst sizes without baseline worrisome features (WF) nor high‐risk stigmata (HRS). The risk of developing WF/HRS/HGD/PC was 1.5‐fold lower in smaller cysts (< 15 mm) than larger cysts (≥ 15 mm). Similarly, a slow growing cyst (growth rate at < 2.5 mm/year) had a lower risk of developing WF/HRS/HGD/PC when compared to faster growing cysts (growth rate at ≥ 2.5 mm/year). When combining cyst size and cyst growth, the risk of developing WF or HRS was lowest for persons with a baseline cyst size < 15 mm and growth < 2.5 mm/year, and highest for cysts ≥ 15 mm and growth ≥ 2.5 mm/year. The absolute risk of developing HGD and PC was lowest for those with a baseline cyst < 15 mm and growth < 2.5 mm/year, and highest for those with a baseline cyst size > 15 mm and growth ≥ 2.5 mm/year. Amongst those patients with a baseline cyst size of < 15 mm, only 1.1% developed HGD or PC and 11% developed WF and HRS after a median surveillance time of 23 months. Similarly, those with cyst growth of < 2.5 mm/year 0.9% developed HGD/PC after a median surveillance of 41 months and 13% developed WF/HRS after a median of 50 months. The findings challenge the current surveillance protocols, including those recently proposed by the revised Kyoto guidelines. In small, slow‐growing PCLs a less intensive surveillance strategy may be acceptable.

Several studies have pointed to a potentially too aggressive policy towards pancreatic resection for perceived IPMN lesions, as the current guidelines result in considerable number of benign lesions found on the final histopathology after pancreatic resection. Further evidence supporting a more cautious approach to pancreatic resection comes from a study by Holmberg et al. [3] who explored survival after pancreatic resection for IPMN with varying levels of dysplasia to early PC (i.e., early‐stage pT1 pancreatic cancer). It is a prudent observation that almost half the patients in this multicenter study who underwent a pancreatoduodenectomy only harboured LGD on histopathology, despite having a cyst size of > 40 mm. While pancreatoduodenectomy is performed with low mortality and reasonable morbidity in most centres, it still remains a high‐risk intervention. Indeed, risk of surgery may outweigh the survival benefit of resecting IPMNs, in particular when the resection is performed for LGD lesions that are unlikely to undergo malignant transformation. The study also showed that almost a quarter of patient undergoing pancreatic resection for LGD lesions developed severe postoperative complications (defined as Clavien‐Dindo grade ≥ 3) and that the overall 1‐year mortality was 2.6%. Furthermore, although the 5‐years OS rate for LGD was 97%, this does not take into consideration the implications on patients' health related quality after major surgery [4].

Taken from the above, we currently face two diagnostic conundrums in the management of small, incidental PCLs. Firstly, are these indeed IPMNs warranting surveillance? In the study by Levink et al., 21% of resection specimens were other cystic aetiologies, including serous cystic neoplasia (SCN) and pseudocysts—lesions that do not warrant surveillance (nor resection) in the first place. The observation of misclassification is supported by a recent systematic review [5] showing that one‐in‐every‐four PCLs are preoperatively misclassified and, furthermore, that use of a preoperative EUS did not reduce the rate of cyst misclassification and, of note, this finding was consistent across health care systems around the world [4]. Hence, the misclassification and low accuracy appear to be a consistent and a universal challenge. Secondly, in radiologically confirmed IPMNs, how well can we differentiate an IPMN with LGD from lesions with HGD or early invasive cancer? Although the risk of HGD/PC in small cysts with slow growth is miniscule, it is not non‐existent and we are likely to miss patients who would benefit from early surgery although the numbers needed to treat are high.

New strategies of risk evaluation, surveillance strategies and indication for surgery are urgently needed, likely with the use of multi‐omics panels defining biological risk of the PCL. A way forward would be genomic analysis of cyst fluid exploring heterogeneity in known mutations to the IPMN lesions, such as GNAS and KRAS which are more prevalent in IPMNs with LGD with convergent evolution in mutations in RNF43 and TP53 leading to development of HGD and invasive cancers [6], a suggestion that is also supported by the revised Kyoto guidelines. This early diagnosis is imperative as firstly survival from resected invasive IPMN is considered better than pancreatic ductal adenocarcinoma [7], with 10‐year survival rates approaching 30% and secondly, the role of adjuvant chemotherapy on survival benefit in invasive IPMN is not fully established [8] and a resection at earlier stages of the diseases undoubtedly confirms survival benefit [3]. The challenge lies in the selection of which patients and lesions to consider for this invasive investigation (biopsies by EUS), the accuracy and representativeness of tissue accrual, and the capacity for mutation testing (usually by next‐generation sequencing platforms) across the health care spectrum.

Although the understanding of surveillance strategies and their outcomes for IPMNs is improving, considerable challenges lie ahead with an increasing volume of incidental and indeterminate PCLS being detected and entering surveillance programmes. The reliance on conventional modalities such as MRI and EUS has the challenge of interobserver variability at small cyst sizes and will meet capacity issues at the current incremental request for surveillance. It is likely future surveillance protocols will be determined by non‐invasive modalities such as next‐generation artificial intelligence and machine learning algorithms AI [9] to identify occult invasive foci or radiologically high‐risk lesions that warrant surveillance. For situations in which a diagnostic conundrum still exists, it is possible that DNA/RNA based next generation sequencing panel of IPMN cyst fluid (or biopsies) will likely determine the need for surveillance [10].

## Conflicts of Interest

The authors declare no conflicts of interest.

## Data Availability

Data sharing not applicable to this article as no datasets were generated or analysed during the current study.
